# The Cancer Survivorship Program at the Abramson Cancer Center of the University of Pennsylvania

**DOI:** 10.1007/s11764-023-01524-9

**Published:** 2024-01-31

**Authors:** Linda A. Jacobs

**Affiliations:** 1 Abramson Cancer Center, Perelman Center for Advanced Medicine, University of Pennsylvania, South Pavilion 10-149, 3400 Civic Center Blvd, Philadelphia, PA 19104 USA; 2 Abramson Cancer Center, University of Pennsylvania, Philadelphia, PA USA

**Keywords:** Cancer survivorship, Follow-up care, Clinical care, Late effects, Program development, Research

## Abstract

The Cancer Survivorship Program was established at the University of Pennsylvania Cancer Center in 2001. The Cancer Center was renamed the Abramson Cancer Center of the University of Pennsylvania in 2002 and the survivorship program was henceforth known as the ACC Survivorship Program. The program was supported from 2001 to 2004 in part by a seed grant from the Lance Armstrong Foundation (LAF). The LIVESTRONG Survivorship Centers of Excellence Network was created by the LAF in 2005 and the ACC Survivorship Program joined the Network in 2007. The seven nationwide Cancer Centers that comprised the Network were supported by the LAF through 2015. A focus on clinical care, research, and education led the development of the ACC Survivorship Program. The program is currently led by an advanced practice provider (APP) and staffed by medical, surgical, and radiation oncology APPs and collaborating oncologists. This program provides care to adult survivors of pediatric cancers, as well as survivors of adult-onset cancers such as breast, genitourinary/prostate, lymphoma, head and neck, gastrointestinal, thoracic, sarcoma, and central nervous system. Research protocols for survivors of specific cancer diagnoses have been developed and have resulted in collaborative research, publications, and conference presentations. Sustaining the ACC Survivorship Program has been challenging despite strong endorsement of services by patients, families, and providers. Challenges include barriers such as cost restraints, changing cancer center priorities, and a reduced oncology workforce, issues experienced across the country that must be addressed in the years to come.

## Introduction

Over the past 50 years, there has been substantial progress in cancer care delivery and survival has improved for children and adults diagnosed with cancer. This has resulted in growing populations of cancer survivors with unique needs and with potential risks for significant medical and psychosocial issues over the course of their lives. The past few decades have seen greater emphasis on provision of optimal follow-up care for cancer patients and long-term survivors living with cancer as a chronic illness. Much of this activity can be attributed to the emergence of informed and assertive healthcare consumers who have unified and coordinated the various components of the early cancer survivorship movement [1, 2] and the 2006 Institute of Medicine consensus report *From Cancer Patient to Cancer Survivor: Lost in Transition* [3].

## History: The Cancer Center at the University of Pennsylvania

The Hospital of the University of Pennsylvania opened in 1874 as the nation’s first teaching hospital for the University’s School of Medicine that was founded in 1765. The Penn Medicine Cancer Center was formally established in 1973 and in June 2002, it was renamed the Abramson Cancer Center (ACC) in recognition of the extraordinary support of Leonard and Madlyn Abramson and family. Located in West Philadelphia, the ACC provides care to those living in Southeastern Pennsylvania and Southern New Jersey and serves the needs of the 12 counties that comprise its catchment area. The population of over 8 million captures approximately 85 percent of patients seen at the ACC, with non-White population comprising approximately 18 percent of cancer patients seen in the West Philadelphia location. This is a matrix cancer center embedded within the University of Pennsylvania Health System, Penn Medicine, and includes community hospitals with in-patient cancer care facilities, in/out-patient rehabilitation, hospice services, and behavioral health (see Table 1). Clinical care is documented in EPIC, the electronic health record system at Penn Medicine.

**Table 1:** Penn Medicine facilities and additional enterprises delivering survivorship care

Penn Medicine’s patient care facilities	Additional Penn Medicine facilities and enterprises
• Hospital of the University of Pennsylvania • Penn Presbyterian Medical Center • Chester County Hospital: Penn Medicine • Lancaster General Health: Penn Medicine • Princeton Health: Penn Medicine • Pennsylvania Hospital: Penn Medicine	• Outpatient community facilities -Radnor -Valley Forge -Cherry Hill -Virtua • Good Shepherd Penn Partners • Penn Medicine at Home • Lancaster Behavioral Health Hospital • Princeton House Behavioral Health

## The Cancer Survivorship Program at Penn Medicine: development and funding

In the 1970s, Anna Meadows, MD, established the first Late Effects Clinic for pediatric cancer survivors at The Children’s Hospital of Philadelphia (CHOP). As a pediatric oncologist and nationally recognized expert in the late effects of cancer treatment, she understood the importance of transitioning survivors into an adult healthcare setting as they aged out of pediatric care. In 2001, in collaboration with the Penn Medicine Cancer Center Director John Glick, MD, Dr. Meadows secured a seed grant (2001-2004) from the Lance Armstrong Foundation (LAF) to establish the first adult cancer survivorship program in the country. In 2001, Linda Jacobs, PhD, CRNP, an oncology and primary care nurse practitioner, was recruited to co-direct the program with Dr. Meadows. When Dr. Meadows retired in 2010, Dr. Jacobs took over as Director. In 2005, a few years after the Penn Medicine Cancer Center was renamed the ACC (2002), The LIVESTRONG™ Survivorship Centers of Excellence Network was created by the LAF to improve the quality of post-treatment cancer care for survivors by expanding knowledge in the field of survivorship. The ACC Survivorship Program joined the Network in 2007 and the seven National Cancer Institute designated Comprehensive Cancer Centers that comprised the Network were supported by the LAF through 2015. These Centers conducted collaborative research projects testing models of care, determining the needs of cancer survivors, and examining the late effects of treatment.

Joseph Carver, MD, a cardiologist with an interest in late effects of cancer treatment, joined the ACC in 2001 and established one of the first Cardio-Oncology programs in the country that has evolved into a training program and referral center. Currently, there are over 600 Cardio-Oncology consults placed each year at the ACC. Dava Szalda, MD, a pediatric oncologist (with a Med-Peds training), joined the team in 2014. She assists with transitioning patients from CHOP to the ACC Survivorship Program and sees survivors in clinic one day each week. Adult survivors of pediatric and young adult cancers of all ages who are not transitioning from CHOP are also followed in the program. Survivors refer themselves or are referred to the survivorship program. In 2001, Meadows and Jacobs identified the need for a project manager, a patient navigator, a behavioral scientist, and a research coordinator to assist with the clinical program development, ongoing research, and establishing education goals of the program.

Since 2015, when the LAF support ended, support for the program has come from the ACC core grant from the National Cancer Institute (NCI), clinical revenue, philanthropy, as well as other grants funded by the NCI, the Department of Defense, the Susan G. Komen Foundation, the Oncology Nursing Foundation, and the Commonwealth of Pennsylvania. One key funded research initiative was the U54 Translational Research on Energetics and Cancer Survivorship Center Grant (2011-2016), a multi-site, multidisciplinary, multi-specialty collaborative effort by the ACC survivorship program team aimed at promoting exercise among breast and testicular cancer survivors [4].

## Clinical care structure

**Fig. 1:**
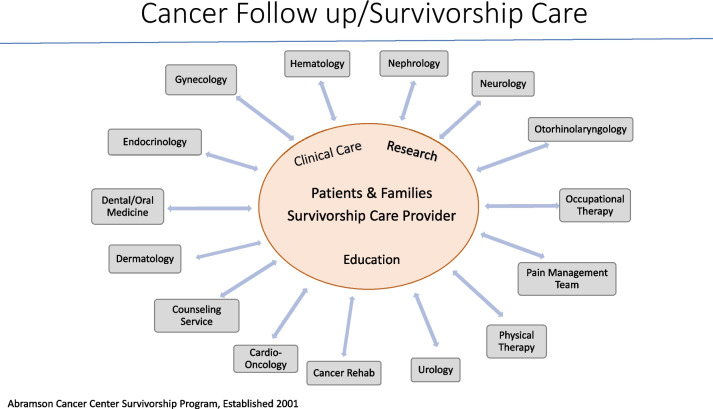
Cancer follow-up/survivorship care

The first few years of program development focused on establishing infrastructure engaging with specialty care colleagues to provide survivorship care (see Fig. 1). This process resulted in rapid access to specialists with minimal wait time for appointments, a successful endeavor that remains in effect today.

A Survivorship Resource Guide was developed by survivorship team members and is revised yearly. This guide outlines services available and contact information for those services. The guide is available in all clinics and easily accessed by patients. Providers and patients use this guide to make referrals to the counseling service, nutrition, financial services, and numerous specialty care providers (e.g., Cardio-Oncology, endocrinology).

The ACC survivorship team has conducted numerous clinical pilot projects to determine the clinical care delivery model best suited specifically for adult survivors of pediatric cancers, as well as models for adult testicular and breast cancer survivors, the first populations of survivors seen at the ACC Survivorship Program. Survivorship clinical care programs for genitourinary/prostate, lymphoma, head and neck, gastrointestinal, and thoracic cancers followed, with care being delivered by APPs collaborating with ACC oncologists. Since the start of the program, patient-reported outcomes (PROs) survivorship questionnaires have been administered at each follow-up visit. These revised over the years and are now sent to survivors prior to their follow-up appointments via the patient portal.

The Program is currently staffed by medical, surgical, and radiation oncology APPs who self-identified to join the program team. The team also includes administrators and physicians from Penn Medicine, the ACC, and affiliate cancer programs. Regular meetings are held where team members provide input on care delivery models at each site, discuss challenges and solutions, in-services on topics of the choice by the team are provided, and best practices for decreasing the number of follow-up patients in the oncology practices debated. The team is currently working with ACC information technology experts and the EPIC team to develop a process that will streamline referrals from providers to survivorship clinics across the Penn Medicine system by developing an electronic order set. In 2011, a few APP members of the program team met with the ACC EPIC team to develop smart phrases that would improve documentation of survivorship care. Improving the clinical encounter notes assisted the ACC in meeting the Commission on Cancer (CoC) as well as the National Accreditation Program for Breast Centers (NAPBC) standards.

All APPs have collaborative practice agreements with every provider to bill for services. In 2017, a financial analysis of the existing breast, gastrointestinal, genitourinary, and thoracic oncology practices was done to determine revenue generated if APPs delivered survivorship care, and more new patient slots were available on physician schedules. Results were impressive in terms of revenue generated from new patient visits and resulting downstream revenue in the Penn Medicine ACC and Health System. Although the profit seen by each disease group was modest, the overall profit to the Health System was upward of 1.9 million dollars per year.

The clinics for adult survivors of pediatric and young adult cancers continue to grow, with over 420 patients seen every year. These clinics also function as centers for long-term adult survivors of lymphoma, sarcoma, neuro-oncology, and other cancers, with most survivors coming to the ACC survivorship program through self-referral or referrals from outside providers. In addition, the ACC has disease-based clinics. Among these, the breast cancer team at the ACC has the largest population of patients and survivors. To meet the need to care for long-term survivors, a full-time APP manages this clinic in collaboration with a senior breast oncologist. This program has been very successful, with over 1000 breast cancer survivors seen in 2022. The ACC also has a busy testicular cancer survivorship program which provides care to approximately 300–400 survivors annually.

Over the last decade, survivorship programs have developed across disease groups in the ACC and affiliates within the medical, surgical, and radiation oncology departments. For example, there are APP-led prostate, head and neck, lymphoma, and rectal cancer survivorship programs based in the ACC radiation oncology department, a multidisciplinary survivorship program based in the Ear, Nose and Throat department at the Penn Medicine Pennsylvania Hospital location, and Lancaster General Hospital is developing a comprehensive survivorship program also led by an APP in collaboration with their Cancer Center director.

Although referrals to specialty services are not formally tracked, patients have expressed overwhelming support for the survivorship program through visit evaluations.

## Research and education

Initial research was conducted in collaboration with the LIVESTRONG™ Survivorship Centers of Excellence Network which included studies evaluating the utility of survivorship care plans [5], metrics to evaluate treatment summaries and survivorship care plans by use of a scorecard [6], and the best practice models of survivorship care [7].

In addition, research protocols were developed during the early years of the survivorship program. As mentioned previously, PROs questionnaire data were collected with each follow-up visit on all patients being seen in the breast, testicular, and young adult survivorship clinics. Consequently, the ACC survivorship program databases contain data from over 600 breast, 700 testicular, and 600 young adult survivors. These data have resulted in poster presentations at national meetings [8–11].

In 2008, the first ACC Survivorship Program retreat was held to assess potential research collaborations across the health system. In 2010, multidisciplinary research findings were presented at a second retreat. In 2022, the multi-disciplinary, multi-specialty scientific retreat sponsored by the ACC Cancer Control Committee presented ongoing research projects and results, including projects on genetic testing, cardiovascular risk factors in cancer survivors, and a study that examined health behaviors and smoking among head and neck cancer survivors [12].

The ACC conducts patient and provider education conferences on a yearly basis on a variety of topics including updates on options for treatment and survivorship care presented by a multidisciplinary faculty. These conferences are open to all disciplines within and outside of the Penn system.

## Challenges and successes

In 2016, the COC released standard 3.3 that included the requirement that a comprehensive care summary and follow-up plan be provided to all individuals receiving curative-intent therapy [13]. The original goal was to facilitate and enhance survivorship care, and an enormous amount of work went into trying to meet the standard. This very challenging effort quickly devolved into focusing more on providing the care plan document and less on delivering the actual cancer survivorship care. However, in 2019, the revised COC standard 4.8 was released [14] and upon review of the requirements, the ACC met the standard requirements with robust programs already in place, a great success for the ACC survivorship program.

The importance of survivorship care plans is recognized at the ACC, and there is an ongoing APP campaign to improve encounter documentation. At the ACC, every follow-up post treatment visit is considered a survivorship visit. Recognizing that a care plan cannot be static and must change with each encounter, care plans are ideally outlined in each visit encounter to reflect ongoing care and future recommendations. Patient charts including the encounter notes and the current care plan are electronically shared with patients/survivors after the visit. This endeavor has proven to be very popular with our patients, survivors, and their other providers. Although progress is slow, many providers have adopted this comprehensive mode of documenting a follow-up visit.

Sources of program funding remain a significant challenge given that the program relies to a large extent on grants for support. We are currently working with the administration advocating for the ACC Survivorship Program to be funded through the Penn Medicine Health System. The 2023 financial analysis, a repeat of the analysis done in 2017 to determine revenue currently generated by new patients entering the system because of follow-up care being provided by APPs, will provide justification for the Health System supporting the survivorship program.

## Future directions

Over the last 22 years, impressive advances in cancer therapy and the management of late effects have resulted in a growing need for strong cancer survivorship programs. Sustaining the ACC Survivorship Program has been challenging despite patients, their families, and providers’ strong endorsement of the services provided. Challenges include barriers such as cost restraints, changing cancer center priorities, and a reduced oncology workforce. However, these issues are being experienced across the country, problems that must be addressed in the years to come to continue to provide necessary, quality care to the growing number of cancer survivors. The ACC is committed to the survivorship program and key program advocates are working with the program team and the Health System to assure that the program continues to grow. In the meantime, cancer survivorship care is recognized as a critical component of cancer care across the Penn Medicine Health System and the ACC. Although challenges will continue, this segment of the cancer care continuum is here to stay.
